# A Comprehensive Review of Modern Cancer Therapies Utilizing Oncolytic Viruses

**DOI:** 10.3390/cells14221825

**Published:** 2025-11-20

**Authors:** Michał Sułek, Agnieszka Szuster-Ciesielska

**Affiliations:** Department of Virology and Immunology, Institute of Biological Sciences, Maria Curie-Skłodowska University, Akademicka 19, 20-033 Lublin, Poland; michal.sulek@mail.umcs.pl

**Keywords:** oncolytic viruses, tumor treatment, immune checkpoint inhibitors, CAR-T cells

## Abstract

Oncolytic viruses (OVs) are gaining traction as advanced tools in cancer therapy. They are distinguished by their ability to destroy malignant cells while sparing normal tissue specifically. In addition to their direct tumor-lysing properties, an essential benefit of oncolytic virus therapy is its capacity to activate both the innate and adaptive immune systems. To enhance these therapeutic actions, many OVs have been genetically engineered to encode immune-modulating factors that reestablish or strengthen antitumor immune responses. Recent studies show that combining OVs with other forms of immunotherapy—such as immune checkpoint inhibitors, CAR-T cells, specific T-cell receptor therapies, or autologous tumor-infiltrating lymphocytes—offers significant advances in cancer treatment. This article reviews how OVs work, discusses strategies to enhance their immunogenicity further, and presents the latest rational combinations of oncolytic viruses with other immunotherapies based on current preclinical and clinical research.

## 1. Introduction

In the 21st century, the overall impact of cancer has been increasing worldwide: both the number of new cases and cancer-related deaths are expected to continue rising. According to the International Agency for Research on Cancer, nearly 20 million new cancer cases were recorded globally in 2022, with projections suggesting this figure could reach 35 million by 2050. In that year, lung cancer became the most frequently diagnosed form of cancer, making up about 2.5 million cases—around 12.4% of all cancers worldwide. Following lung cancer, the most common types included breast cancer in women (11.6%), colorectal cancer (9.6%), prostate cancer (7.3%), and stomach cancer (4.9%). Lung cancer also accounted for the highest number of cancer-related deaths, estimated at 1.8 million (18.7%), followed by deaths from colorectal (9.3%), liver (7.8%), breast (6.9%), and stomach (6.8%) cancers. Breast cancer was the leading diagnosis among women, while lung cancer was most prevalent and deadliest among men [1]. Demographic changes—such as population aging, longer average life expectancy, and lifestyle factors like smoking, obesity, and poor nutrition—are major contributors to these trends. Effective prevention strategies could therefore prevent millions of future cases and save numerous lives, resulting in substantial social and economic gains for nations worldwide in the decades ahead.

Modern cancer therapies include traditional methods like surgery, chemotherapy, and radiation, along with newer approaches such as targeted therapy, immunotherapy (including checkpoint inhibitors and CAR T-cell therapy), and hormone therapy [2,3]. These advancements aim to target cancer cells more precisely, minimizing harm to healthy tissue and potentially leading to improved treatment outcomes.

Over the last twenty years, a crucial discovery in cancer research has revealed that tumors can create protective barriers akin to force fields against the immune system. As a result, significant efforts are being focused on dismantling these defenses, such as using viruses that specifically target cancer cells. These viruses, known as oncolytic viruses (OVs), thrive within cancer cells while sparing healthy cells. They multiply rapidly, causing the cancer cells to burst open and exposing their contents to the immune system. Not only do these viruses disrupt cancer naturally, but they can also be engineered to deliver additional genes into cancer cells, improving the treatment’s effectiveness in eliminating tumors.

In recent years, integrating oncolytic viruses with other immunotherapies—such as immune checkpoint inhibitors, CAR-T therapies, antigen-targeted T-cell receptors, and autologous tumor-infiltrating lymphocyte treatments—has led to notable progress in cancer therapy. This review summarizes the early history of discovering and testing native OVs, examines the core biological functions of OVs, explores methods to enhance their antitumor immune effects through genetic modification, and highlights the impact of combining OVs with other immunotherapeutic strategies as shown in contemporary preclinical and clinical studies.

## 2. History of Using Oncolytic Viruses

Historical observations suggest that viral diseases might have played a positive role in cancer remission since the late 1800s. Interestingly, at that time the causes of these diseases were still unknown, and only the presence of infectious particles smaller than bacteria was recognized. Martinus Beijerinck was the first to use the term “*contagium vivum fluidum*” to describe the small and unknown agent causing tobacco mosaic disease. As a result, the earliest reports in this area were not intentional descriptions of the beneficial effects of oncolytic viruses but accounts of illnesses caused by them.

**Figure 1:**
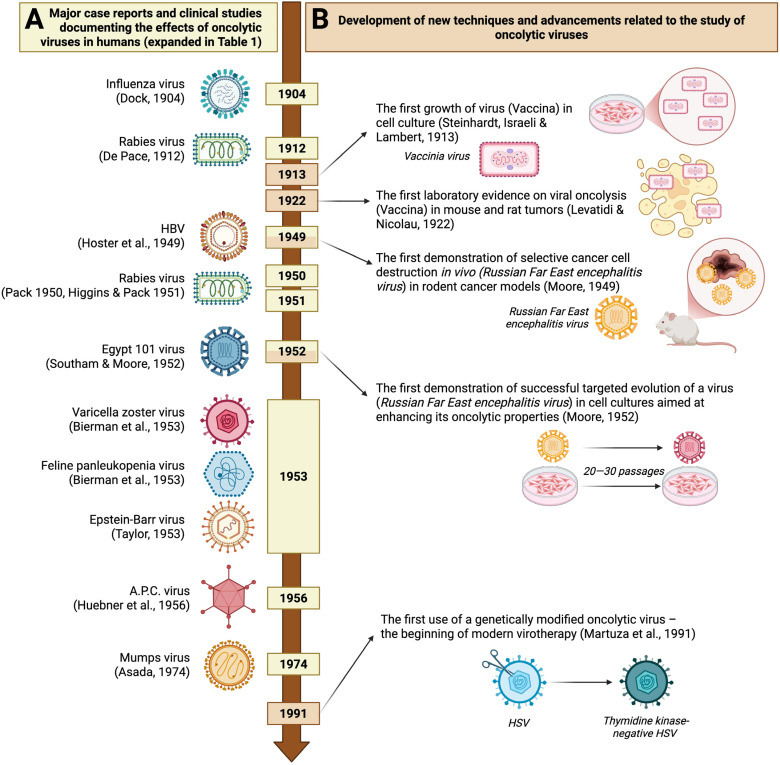
Significant milestones in the development of early oncolytic virotherapy. Side A (yellow) displays major case reports and clinical studies, indicating the virus used and the author of each study (details expanded in Table 1). Side B (light brown) highlights the development of new techniques and advancements that contributed to improving early virotherapy, along with the author(s) of the relevant studies. HBV—hepatitis B virus; A.P.C.—adenoidal—pharyngeal—conjunctival (adenoviruses); HSV—herpes simplex virus [4,8,9,10,11,12,13,14,15,16,19,22,24,25,26]. (created with BioRender.com, accessed on 19 October 2025).

One of these early reports is the case described by Dock, which details the medical history of a 42-year-old woman with myelogenous leukemia (Figure 1, Table 1). During her clinical observation from 1896 to 1897, she contracted influenza. As a result, she experienced a several-week remission of leukemia symptoms, characterized by a significant decrease in leukocyte count (from 367,000 cells/µL to 5000 or less), a reduction in the size of her enlarged liver and spleen, and an overall improvement in her condition. However, after the remission, her health deteriorated, eventually leading to her death a few months later [4].

In the following years, efforts to find new anticancer compounds still did not lead to a major breakthrough. Due to the lack of an effective cure, the idea that infections could occur alongside cancer and be linked to periods of temporary remission became another topic of speculation [5]. Most observations regarding the anticancer effects of viral infections have been made in patients with blood cancers, as their weakened immune systems allowed the viral infections to persist and show effects that would otherwise be hidden [6].

Hoster et al. reported a beneficial effect of viral hepatitis, which led to long-lasting remission periods of Hodgkin’s disease in two men in their thirties (Figure 1, Table 1). In the described clinical cases, where accidental viral transmission occurred through blood transfusion and the yellow fever vaccine, remission periods of about four years were observed, preceded by noticeable jaundice. However, it is worth noting that these patients also received standard therapy (X-ray and/or nitrogen mustard treatment) either before or during the viral hepatitis-induced remission. Therefore, the beneficial effect may have resulted from a synergistic interaction between the viral infection and conventional treatment [6].

Later, Wintrobe et al., in their work focusing on the beneficial use of hormone therapy involving corticotropin, documented a temporary remission in a 4- to 5-year-old girl with leukemia who contracted chickenpox during her illness. Interestingly, the remission occurred while the girl was receiving aminopterin, whose therapeutic effect had already begun to fade. The beneficial effect of the viral infection was evident as a noticeable drop in leukocyte count and a reduction in spleen size. After the remission period, an experimental corticotrophin therapy was introduced, which, although followed by a temporary remission, did not produce a long-lasting effect [7].

A key study focusing solely on how acute infections impact leukemia patients, leading to remission periods, was presented by Bierman et al. (Figure 1, Table 1). Among the cases discussed was a 4-year-old boy who developed chickenpox, which then caused a one-month remission of leukemia. This remission, similar to what was observed in earlier studies, was marked by a reduction in liver and spleen size, as well as a decrease in leukocyte count [8]. Based on documented cases of cancer remissions following incidental viral infections like influenza or chickenpox, experimental therapies using samples with live viruses were initiated.

The earliest report of virotherapy is generally credited to N. DePace, who in 1912 observed the regression of cervical carcinoma in a patient after rabies vaccination, suggesting that the injected attenuated virus might serve as an oncolytic agent (Figure 1, Table 1) [9]. However, it was not until decades later that the intentional, experimental use of virus particles began as part of broader clinical trials for cancer treatment. These studies were often carried out by the same research teams that had previously reported on the therapeutic effects of viruses in cases of unintentional infections. Building on earlier observations of potential benefits from chickenpox, Bierman et al. inoculated six children with leukemia using throat washings from individuals who had contracted chickenpox (Figure 1, Table 1). However, these studies failed to yield reliable data since four of the children did not develop clear signs of varicella, and the remaining two died during the incubation period [8]. Continuing their experimental approach to oncotherapy, Bierman et al., inspired by observations of leukopenia in cats infected with feline panleukopenia virus, decided to investigate whether inoculation with a virus suspension could help reduce the overproduction of leukocytes in leukemia patients. Among six children inoculated with a feline spleen suspension, only one exhibited a noticeable response to the infection—a decrease in leukocyte count—that led to a one-month remission of both hematological and clinical symptoms (Figure 1, Table 1) [8].

Around the same time, experimental oncolytic therapies using different viruses gained popularity. Building on DePace’s findings, Pack reported his initial observations on the experimental use of the rabies vaccine for melanomatosis (Figure 1, Table 1). In his report, he described a case of a woman with malignant melanoma who, after receiving a series of rabies vaccinations in 1943, experienced a latency period of over five years between the initial appearance of metastases, which dated back to 1942. Inspired by these results, he started a clinical trial involving 12 private patients who received a series of rabies vaccines. According to the published clinical notes, positive effects of the attenuated virus inoculation were seen in two patients. One showed flattening and loss of firmness in numerous existing skin metastases. The second patient experienced a reduction in the size of a liver previously infiltrated by cancerous cells due to distant metastases. In later clinical notes, Higgins & Pack reported “regressive changes” among eight of thirty melanomatosis patients following rabies vaccination (Table 1) [10]. The urgent need for an effective cancer treatment, media pressure on scientists, and enthusiasm for experimental research are reflected in Pack’s concluding remarks, which we quote: “I am motivated in publishing this incomplete and unsuccessful story at this premature time by the unfortunate fact that news of this experiment has become widely disseminated. I have been besieged by numerous telegrams, letters, and telephone calls requesting more specific information. In self-protection and wishing to prevent the hope this false rumor has sparked, I have reported the experiences of my colleagues and myself to date.” [11]

The problem of ineffective therapies was especially urgent in cancers where experimental chemotherapy drugs like urethane or nitrogen mustards had no effect or even harmed the patient, as seen in acute leukemia. Taylor, inspired by observing clinical remission of acute leukemia in an adult patient after an incidental glandular fever infection, conducted a study in which five patients with acute leukemia were injected with serum containing Epstein-Barr virus (Figure 1, Table 1). In three of the five patients, symptoms of viral infection appeared, followed by periods of cancer remission. The other two patients showed no signs of glandular fever, and their cancer progressed rapidly and continually worsened [12].

A well-known and frequently cited example of one of the first successful clinical trials involving the use of an OV was conducted by Hoster et al. (Figure 1, Table 1). After observing remissions of Hodgkin’s disease in patients accidentally infected with the hepatitis virus, they continued with further clinical studies, including intentionally administering the virus to 21 cancer patients. Of these, 13 developed noticeable symptoms of viral infection, and 7 showed visible improvement—mainly indicated by a normalized leukocyte count, pain relief, and a reduction in tumor size. In patients who did not develop hepatitis, no remission of Hodgkin’s disease was observed [13].

An important step in studying OVs was the development of research models that allowed investigation of their activity both in vitro and in vivo, excluding humans. One of the pioneers in using human cell cultures and rodents as research models for oncolytic virus properties was Moore (Figure 1). She documented that infection with the Russian Spring-Summer Encephalitis virus in mice can inhibit various tumors [14,15]. Her studies gained recognition from Southam, who initiated a series of clinical trials using the vaccinia virus, Newcastle disease virus, West Nile virus, Ilhéus virus, Bunyamwera virus, and finally the Egypt 101 virus—a strain of early-passaged West Nile virus. It was specifically the studies involving the latter that produced remarkable results, making them among the most significant in early virus oncology (Figure 1, Table 1). Thirty-four patients with neoplastic diseases unresponsive to other treatments were injected with a single dose of the Egypt 101 virus. Twenty-seven showed signs of infection, four experienced significant remission, and five exhibited slight improvements, also suggesting a beneficial effect of the virus injection. Histopathological analyses of tissues confirmed the presence of the virus in tumor tissue. In five patients, the virus was detected in the tumor tissue, and in another five, it was preferentially concentrated in the tumor tissue compared to normal tissue. This was important, as the virus was never directly injected into the tumor mass [16].

One potential issue with using OVs, discussed among the authors of the studies cited earlier, was the development of immunity after the first exposure to the virus. This led to a decrease or loss of its beneficial effect in subsequent inoculations or a complete lack of effectiveness of the initial treatment if the individual had previously been infected with a specific virus and already had antibodies. Therefore, despite limited information on how OVs work, efforts have been made to test various viruses, as shown by the scientific studies referenced so far. At that time, oncologists also became interested in a newly discovered group of adenoidal-pharyngeal-conjunctival agents linked to respiratory infections. The cytopathic properties of these adenoidal-pharyngeal-conjunctival (A.P.C.) agents (before they were officially recognized as a new family of viruses, according to modern taxonomy—adenoviruses) were documented in various tissue cultures, including HeLa cells [17]. Following these observations, and after the official classification of these agents as viruses, studies began to examine their potential for cancer treatment in vivo (Figure 1, Table 1) [18]. Huebner et al. injected adenoidal-pharyngeal-conjunctival virus into forty patients with cervical carcinoma and observed necrosis and “cavity formation” in the central part of the pelvic tumor in 26 patients. Notably, the viral effect was limited solely to tumor tissues, and its selective action did not cause lytic effects in healthy tissues within the pelvic area. The inoculations did not produce any harmful effects related to acute infections, highlighting that the use of this new viral family—adenoviruses—could be a breakthrough in the emerging yet promising field of oncolytic virus therapy [18,19].

This was already the fifth major clinical study documenting the oncolytic effects of various viruses, including RNA viruses from the families *Rhabdoviridae*, *Flaviviridae*, *Hepadnaviridae*, and *Orthoherpesviridae*, as well as DNA viruses from the *Adenoviridae* family. New viral species were described, such as the measles virus, first isolated in 1954 by Enders & Peebles, which also documented periods of remission in cases of spontaneous infections in patients with blood cancers [20,21]. However, at that time, these discoveries did not lead to a significant clinical breakthrough, and a major study marking the end of the early era of OVs, before the rise of genetic engineering, was described by Asada, who summarized the effects of mumps virus therapy.

This was by far the most comprehensive report on the experimental use of any OV. Asada summarized clinical experiments involving 90 cancer patients with various terminal cancers (Figure 1, Table 1). The most common types of cancer among them were gastric (33), pulmonary (10), uterine (9), and cutaneous (6). The virus was administered to patients based on tumor location, including intravenous, oral, external, and nasal routes, and the documented effects were remarkable. Of the 90 patients, 37 showed significant improvement, such as tumor reduction or complete disappearance. An additional 42 patients experienced less dramatic but still observable effects, such as slowed tumor growth and overall physical improvements like increased appetite, weight gain, and reduced pain [22].

This was the last major study documenting the use of wild-type viruses in cancer therapy. As chemotherapy and radiotherapy became more popular as reliable treatments, interest in using OVs declined. In its current form, this therapy posed more risks and limitations than conventional methods [23]. The use of live viruses was associated with infections, which could be especially risky for patients already affected. Additionally, contracting such an infection often led to the development of immunity, requiring different viruses for subsequent treatments to maintain the therapeutic effect.

Studying the complex interactions within the “immunological triangle”—which includes the human immune system, the tumor microenvironment, and tumor immunology—as well as the replication and evolution of viruses, has been (and still is) very challenging. However, it is essential for understanding the specific mechanisms behind the safety and mode of action of OVs, which were previously unknown. Another challenge was learning how to specifically ‘target’ the tested viruses to cancer cells. Pioneering work was done by Moore, who documented that continuous passaging of the Russian encephalitis virus in Sarcoma 180 cancer cells transplanted into mice increased its ability to kill that tumor compared to the original virus (Figure 1). This was significant because, after 20 to 30 passages, the virus could even destroy the entire transplanted tumor, compared to about 40% destruction achieved in that time by the original, “non-adapted,” strain [15]. Although a ‘non-deliberate’ genetic approach to improving the virus’s selectivity was observed during this period, it was still insufficient. There were no tools available that would allow scientists to intentionally enhance the virus in a controlled way, rather than relying on the virus itself to adapt.

The revival of interest in OVs dates back to the early 1990s. The emergence of a new scientific field, genetic engineering, paved the way for genome editing, which also found applications in virology. In 1991, Martuza et al. conducted pioneering research in which, for the first time, a genetically modified OV was used in oncolytic therapy (Figure 1). They utilized a mutant of herpes simplex virus-1 with a mutation introduced to eliminate the gene responsible for thymidine kinase synthesis—an enzyme primarily responsible for the virus’s neuropathogenic effects on normal brain cells. Martuza’s study showed that the HSV-1 mutant, attenuated for neurovirulence, retained its oncolytic properties against glioma monocultures and human xenografts in mice [24]. Following the development of the first genetically modified oncolytic virus, a period of rapid growth in modern virotherapy began (Figure 1, Table 1).

**Table 1:** Summary of major case reports and clinical studies documenting the effects of oncolytic viruses in humans.

Year	Authors	Virus (Family)	Disease	Study Detail	Outcome	Significance
1904	Dock, G. [4]	*Influenza viruses* (*Orthomyxoviridae*)	Leukemia	Case report describing a female patient who contracted influenza during her course of leukemia.	Several weeks of remission observed, with an overall improvement in well-being, followed by a reduction in the size of the liver and spleen and a decrease in leukocyte count.	The first significant study documenting the oncolytic effect of viral infection.
1912	De Pace, N. [9]	Attenuated *Lyssavirus rabies* (*Rhabdoviridae*)	Cervical cancer	Clinical study (8 patients) preceded by a case report demonstrating the oncolytic effect of an attenuated rabies vaccine.	Regression of cervical carcinoma in a patient who received Pasteur treatment after a dog bite, leading to the intentional use of an attenuated rabies virus in other patients.	The first recorded clinical trial of virotherapy.
1949	Hoster, H.A.; Zanes, R.P., Jr.; Von Haam, E. [13]	Hepatitis B virus (*Hepadnaviridae*)	Hodgkin lymphoma	Clinical study (21 patients), preceded by two case reports, showed that accidental hepatitis B virus infection had a beneficial effect on Hodgkin’s disease.	13 of 21 patients developed viral symptoms, of whom 7 showed improvement (normalized leukocyte count, pain relief, tumor reduction). Patients without hepatitis symptoms showed no remission.	The first major clinical study using a hepatitis virus and showing a clear and significant therapeutic effect.
1950	Pack, G.T. [11]	Attenuated Lyssavirus rabies (*Rhabdoviridae*)	Melanoma	Clinical study of 12 private patients	Regressive changes were seen in 2 of 12 patients: loss of firmness in multiple existing cutaneous metastases (patient 1) and a reduction in the size of a liver previously affected by distant metastases (patient 2).	The first major series of clinical studies using attenuated live rabies virus (Harris rabies vaccine) for treating melanoma.
1951	Higgins, G.K.; Pack, G.T. [10]			Clinical study of 30 patients	Regressive changes observed in 8 of 30 patients with melanomatosis.	
1952	Southam, C.M.; Moore, A. E. [16]	Egypt 101 virus [early passaged West Nile virus] (*Flaviviridae*)	Neoplasm diseases, with the majority being large bowel adenocarcinoma (26%), epidermoid carcinoma (21%), and breast cancer (9%).	Clinical study of 34 patients	27 out of 34 patients were successfully infected. Among these, 4 out of 27 experienced transient regression of tumor growth; 5 out of 27 showed a probable viral effect on tumor growth inhibition; and 14 out of 27 demonstrated oncotropism, with the virus showing a preference for tumor tissue over healthy tissue in 5 out of 27 cases.	The first major clinical study using Flaviviridae viruses to target multiple neoplastic diseases. Demonstrates oncotropism without direct intratumoral viral administration.
1953	Bierman, H. R. et al. [8]	Varicella zoster virus (*Orthoherpesviridae*)	Leukemia	Clinical study involving 6 children, following a case report of a 1-month leukemia remission in a child who accidentally developed chickenpox.	2 of the 6 children died during the incubation period, and the other four did not develop varicella (likely due to passive immunoglobulin transfer during blood transfusion).	First attempts at virotherapy using varicella and feline panleukopenia viruses against leukemia.
		Feline panleukopenia virus (*Parvoviridae*)		Clinical study of 6 children	2 out of 6 children died before showing any signs of viral infection; of the remaining four children who were injected with the virus, one experienced a one-month remission, characterized by a drop in leukocytes and clinical improvement.	
1953	Taylor, A. W. [12]	Epstein–Barr virus (*Orthoherpesviridae*)	Leukemia	Clinical study of 5 patients preceded by observation of spontaneous remission after accidental glandular fever infection.	3 out of 5 patients showed a regression of leukemia symptoms, including a decrease in monoblast count and an increase in red blood cells and platelets, following the onset of glandular fever. In the remaining two patients, there was no evidence of viral infection.	First attempt at virotherapy using Epstein-Barr virus against leukemia. Documented exceptionally high effectiveness of the viral infection in inducing cancer remission.
1956	Huebner R.J. et al. [19]	Adenoidal-pharyngeal-conjunctival virus (*Adenoviruses*)	Epidermoid carcinoma of the cervix	Clinical study of 30 patients	26 out of 40 virus injection cases caused local necrosis exclusively within the cancer tissue. The effect’s strength was inversely proportional to the amount of antibodies in the patients’ serum.	First attempt at virotherapy using a newly discovered family of viruses, first described in 1953.
1974	Asada, T. [22]	Mumps virus (*Paramyxoviridae*)	Terminal cancers of various types, mostly gastric (37% of cases), followed by lung (11%) and uterine cancers (10%)	Clinical study of 90 patients	Out of 90 cases, 37 showed tumor disappearance or more than 50% size reduction; 42 demonstrated tumor growth inhibition, regression, or overall health improvement; 11 exhibited no response, and 7 experienced adverse effects such as transient high fever and profuse bleeding.	The largest and most comprehensive clinical study on non-genetically engineered oncolytic viruses, including extensive visual and histological documentation of the observed changes.

## 3. Types of OVs

**Figure 2:**
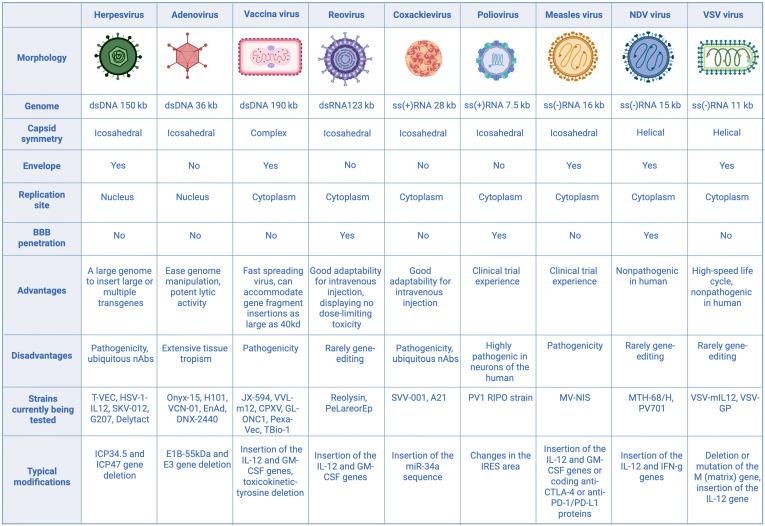
Characteristics of major oncogenic viruses, their advantages and disadvantages for application [27]. NDV—Newcastle Disease Virus, VSV—Vesicular Stomatitis Virus, BBB—Blood–brain barrier, *nAbs*—neutralizing antibodies, (+) or (−) indicate positive— and negative—sense ssRNA, respectively (created with BioRender.com, accessed on 31 May 2025).

Oncolytic viruses originate from viruses that contain either single- or double-stranded DNA or RNA, depending on the type of nucleic acid. The most common types of viruses in OV products are ssRNA and dsDNA viruses, with exceptions such as reovirus (dsRNA) and parvovirus (ssDNA). Double-stranded DNA viruses mainly include adenovirus, vaccinia virus, and herpesvirus. In contrast, single-stranded RNA viruses are divided into two categories: positive-sense (such as coxsackievirus and poliovirus) and negative-sense (including measles virus, Newcastle Disease virus, and vesicular stomatitis virus) (Figure 2).

### 3.1. Herpesvirus

Herpes simplex virus (HSV) is a lipid-enveloped virus containing a double-stranded DNA genome, which is housed within a nucleocapsid and surrounded by a tegument layer. There are two primary serotypes—HSV-1 and HSV-2 [28]. With a large genome of at least 150 kilobases and a complex structure, HSV is well-suited for the incorporation of sizable DNA inserts and multiple foreign genes [29]. Its viral envelope features four major glycoproteins—gB, gD, gH, and gL—that enable the virus to bind to a range of cellular receptors [30]. During infection, the viral envelope merges with the host cell membrane, allowing the nucleocapsid to reach the nucleus [31]. The viral genetic material then enters the cytoplasm and moves into the nucleus, where gene transcription begins. HSV genome organization ensures tight regulation of viral gene transcription and protein production. Viral proteins are grouped as immediate-early, early, or late depending on their order of synthesis, and targeting these proteins for gene modification is a common strategy [32]. As a lytic virus, HSV can infect and destroy a broad spectrum of cancer cells, multiply rapidly, and efficiently disseminate within tumor tissues [33].

To enhance the safety of oncolytic HSV therapy, antiviral agents such as Acyclovir can be administered to neutralize viral pathogenicity [34]. Although more than half of people carry neutralizing antibodies against HSV, the virus can evade host immune defenses through various immune evasion strategies, making it a valuable template for development as an oncolytic virus vector [35]. HSV-1, in particular, is widely used in oncolytic virotherapy research. Notable HSV-1-based vectors include Talimogene laherparepvec (T-VEC)—the first oncolytic virus approved by the FDA in 2015 for advanced melanoma—along with G207 and G47Δ [36,37,38]. HSV-2 has also emerged as a promising candidate for further investigation in this field. An oncolytic HSV-2 known as OH2 has recently entered some clinical studies for melanoma (NCT05868707), solid tumors (NCT03866525, NCT04386967), central nervous system tumors (NCT05235074), and advanced bladder cancer (NCT05248789).

### 3.2. Adenovirus

Adenovirus is a non-enveloped virus roughly 90–100 nm in diameter, containing a double-stranded DNA genome of about 26–45 kb that is packaged within an icosahedral capsid composed of hexon trimers and penton bases (PB) [39]. The fiber protein’s N-terminal region connects to the penton base, while its C-terminal segment plays a crucial role in binding to cellular receptors, making it a prime site for modifications aimed at targeted therapy [40]. Of the 57 identified adenovirus serotypes, Ad2 and Ad5 (subgroup C) are the most widely used in oncolytic adenovirus studies [41]. Most oAds gain cell entry through interactions with the coxsackievirus and adenovirus receptor (CAR), although certain group B and some group D types use CD46 instead [42]. Upon cell entry via receptor-mediated endocytosis, the viral capsid disassembles, escape the endosome, and then migrate along microtubules to the nuclear envelope. Here, the viral genome is transported into the nucleus [43,44]. For Ad2 and Ad5, early genes *E1A* and *E1B* are critical in activating subsequent transcription and replication of viral genes [45]. E1A contains a conserved region 2 that disrupts retinoblastoma (Rb) protein binding to E2F transcription factors, driving quiescent cells into the S-phase. *E1B* encodes two proteins, 19 kDa and 55 kDa, that together prevent apoptosis after infection and prolong the duration of viral replication. The E1B 55 kDa protein interacts with p53 to promote its degradation, while the 19 kDa E1B form acts to block apoptosis [46,47,48]. Adenoviruses are frequently used for gene therapy and oncolytic applications due to their high viral yield, ease of genetic manipulation, and robust lytic capacity [49]. Nonetheless, because adenoviruses tend to infect a broad range of cells, advancing safety depends on improving selective replication in tumor tissue. One common strategy is the deletion of *E1A* and *E1B* genes, resulting in replication-defective adenoviral vectors for controlled or safer applications [50]. Onyx-15 was the prototype and earlier version from which the Chinese H101 (Oncorine) was later developed. Both are replicating type 5 adenoviruses with a 55 kDa deletion of the *E1B* gene (linked to p53 inhibition), and H101 has an additional deletion in the *E3* region. Onyx-015, H101, and another modified adenovirus CG 0070 have shown promising results in clinical trials [51,52,53,54].

### 3.3. Vaccinia Virus

The vaccinia virus (VV) is a lipid-enveloped, double-stranded DNA virus that belongs to the Orthopoxvirus genus in the *Poxviridae* family [55]. It has a genome of roughly 190 kb and measures about 70–100 nm in diameter, offering ample capacity for insertion and robust expression of large foreign genes [56]. VV encodes critical enzymes required to initiate viral transcription post-infection. The viral core, as well as steps required for replication and virion formation, are located in cytoplasmic mini-nuclei enveloped by the endoplasmic reticulum (ER) [55,56]. Targeting specificity of VV is strongly influenced by the thymidine kinase (TK) gene, which is essential for viral replication and is commonly upregulated in cancer cells but expressed at low levels in normal cells. To exploit this, scientists have engineered TK-deficient strains of VV that are able to replicate in malignant tissue selectively [57]. In addition, VV produces proteins that activate the host cell’s EGFR-RAS pathway, further enhancing TK production. Advantages of the vaccinia platform include its rapid replication cycle, highly efficient intra-tumoral spread, a capacity to accommodate DNA inserts up to approximately 40 kb, and a thoroughly characterized genome—attributes inherited from its use in smallpox vaccines [58]. Among oncolytic VV vectors, JX-594 is especially notable for its resistance to antibody- and complement-mediated inactivation, supporting its potential for systemic (intravenous) delivery [59].

### 3.4. Reovirus

Reovirus (RV) is a non-enveloped virus with double-stranded RNA that is approximately 23.5 kb long. It belongs to the *Reoviridae* family and can infect a variety of hosts, including fungi, plants, fish, reptiles, birds, and mammals. Its RNA is divided into 10 segments [55].

Reovirus enters cells primarily through receptor-mediated endocytosis, attaching to junctional adhesion molecule A, which serves as its entry receptor [56]. Since this receptor is upregulated in numerous cancers—such as breast [57], non-small cell lung cancer [58], diffuse large B-cell lymphoma [59], and multiple myeloma [60] reovirus is well-suited for use as an oncolytic virus targeting these tumor types. Research indicates that successful RV replication and progeny formation depend on the Ras signaling pathway in host cells [61]. Additionally, RV can induce cell apoptosis via the Ras/RalGEF/p38 pathway [62], allowing it to specifically target tumor cells with Ras overexpression. There are three main reovirus serotypes, with the type 3 Dearing strain—known as Reolysin—being widely adopted as a clinical oncolytic agent [63]. This strain is especially suitable for intravenous delivery, showing robust antitumor effects in studies without eliciting significant toxicity or irritation [64]. Currently, Reolysin is the most prominent oncolytic RNA virus in cancer therapy, having been tested in numerous clinical trials both as monotherapy and in conjunction with other treatments [65].

### 3.5. Coxsackievirus and Poliovirus

Coxsackievirus and poliovirus are members of the *Picornaviridae* family and are non-enveloped viruses with an icosahedral capsid, which can be observed using electron microscopy. Their replication occurs in the cytoplasm, preventing integration of foreign genetic material [66]. For cellular entry, coxsackievirus binds to molecules such as DAF and ICAM-1, which are frequently overexpressed in certain cancers—like melanoma, multiple myeloma, and breast cancer [67] While antibody neutralization remains a barrier for coxsackieviruses, serotypic differences make cross-reactivity rare. The Seneca Valley Virus (SVV), especially the SVV-001 variant, is nonpathogenic in humans but has been demonstrated to infect neuroendocrine tumors [68]. Poliovirus, though highly neurotropic and pathogenic in human anterior horn cells, poses toxicity issues that must be addressed for therapeutic purposes. Gromeier and colleagues engineered a poliovirus variant (PV1 RIPO) by substituting its internal ribosomal entry site with that of human rhinovirus type 2, thereby directing the virus to target glioblastoma multiforme—leveraging the overexpression of the poliovirus receptor CD155 on glioma cells [69].

### 3.6. Measles Virus and Newcastle Disease Virus (NDV)

Measles virus (MeV) and Newcastle disease virus (NDV) belong to the *Paramyxoviridae* family. They have relatively large viral particles but a relatively short RNA length [70]. MeV infects host cells by binding to three receptors: CD46, signaling lymphocyte-activation molecule (CD150), and poliovirus receptor-like 4 (PVRL4) [71]. The CD150 receptor is often highly expressed in hematologic malignancies, while CD46 expression is common and elevated in many tumor types, thus providing MeV with a natural tendency to infect cancer cells [72]. However, CD46 is present at basal levels in normal tissues as well, meaning it is not a tumor-restricted marker [71]. MeV is considered a promising therapeutic candidate due to its lack of dose-limiting toxicity and innate selectivity toward tumor cells (oncotropism) [73]. NDV, by contrast, attaches to cancer cells via its hemagglutinin-neuraminidase protein, which interacts with sialic acid residues on the cell surface. The viral F protein is then activated, enabling membrane fusion and genome entry into the cytoplasm of the host cell [74]. NDV’s antitumor action primarily stems from its intrinsic cancer-killing properties and the susceptibility of malignant cells to viral infection. Extensive research demonstrates that NDV efficiently targets a broad range of human tumor cell lines from all three germ layers [75], including cells from colorectal, gastric, pancreatic, bladder, breast, ovarian, renal, lung, laryngeal, and cervical cancers, as well as glioblastoma, melanoma, pheochromocytoma, various lymphomas, fibrosarcoma, osteosarcoma, and neuroblastoma, while sparing normal cells [76,77,78]. One of the best-documented lytic strains of NDV is MTH-68/H, which has been used in clinical trials and compassionate-use therapies for patients with glioblastoma multiforme, glioma, and other solid tumors [75,79]. Moreover, in the past, a lytic strain with high replicative capacity and potent oncolytic activity, PV701, was tested in phase I/II clinical trials as an intravenous and local therapy [79].

### 3.7. Vesicular Stomatitis Virus

Vesicular stomatitis virus (VSV) is a member of the *Rhabdoviridae* family and is classified as a negative-sense RNA virus. Its genome is relatively compact, consisting of about 11,000 nucleotides and encoding five different viral proteins. The viral glycoprotein (G protein) allows VSV to attach to and fuse with host cells via the widely expressed low-density lipoprotein receptor. Following attachment, the virus is internalized through receptor-mediated endocytosis and subsequently transported into endosomes [80]. VSV replication is more efficient in cancer cells than in normal cells, largely because cancer cells frequently exhibit defective interferon signaling pathways, that normally help restrict viral spread [81].

OVs can be divided into (i) naturally attenuated viral strains and (ii) genetically modified viral vectors.

## 4. OVs—Naturally Attenuated Viral Strains

Oncolytic viruses are engineered or selected to attack tumor cells by leveraging the same signaling pathways, cell surface receptors, and mechanisms exploited by cancer for survival—such as immune escape, altered surface proteins, and oncogenic signaling. Creating effective cancer therapies remains challenging, but this can be addressed by deploying viruses that naturally prefer to infect specific cell types or by reengineering them for enhanced specificity. Many oncolytic viruses are chosen because they are naturally drawn to cell surface molecules that are common or upregulated on tumor cells. As one example, T-VEC utilizes surface proteins like HVEM (*herpesvirus entry mediator*), nectin-1, and nectin-2, all of which are often found at higher levels in various tumors, making malignant cells more vulnerable to herpesvirus infection relative to normal tissue [82]. Another approach uses viruses that target cancer cell markers, such as CD46; tumors frequently express this molecule to evade complement-mediated immunity, allowing the measles virus (Edmonston strain) to infect cells via CD46 and selectively kill cancer cells with high receptor expression [83]. Cancers such as breast, multiple myeloma, and melanoma often upregulate ICAM-1 and DAF, which can be exploited by coxsackievirus strains like coxsackievirus A21 (CAVATAK) [67,84]. Poliovirus recognizes specifically CD155, a marker commonly overexpressed in cancer, helping the virus evade innate immune responses—especially antitumor natural killer (NK) cell activity [85].

Oncolytic viruses mainly exert their effects by directly destroying tumor cells through cell lysis. Some of these viruses take advantage of blocked or defective apoptotic pathways in cancer to redirect malignant cells toward alternative forms of cell death. For instance, parvovirus H-1PV (ParvOryx) has undergone phase II clinical testing for metastatic, inoperable pancreatic ductal adenocarcinoma (NCT02653313), as well as for progressive primary or recurrent glioblastoma multiforme (phase II, NCT01301430). Recently, the first clinical trial of parvovirus in patients with progressive, recurrent glioblastoma was completed. Many cancers, such as glioma and pancreatic ductal adenocarcinoma, actively block programmed cell death signals, keeping apoptotic pathways suppressed even during viral infection. To overcome this, H-1PV induces tumor cell death via cathepsin-dependent mechanisms rather than relying on apoptosis [86,87]. This immunogenic, non-apoptotic form of cell death stimulates the release of interferon-gamma (IFN-γ) and various inflammatory cytokines and exposes tumor-specific neoantigens generated by oncolytic activity. These factors cooperate to generate strong antitumor immune responses [87,88].

**Figure 3:**
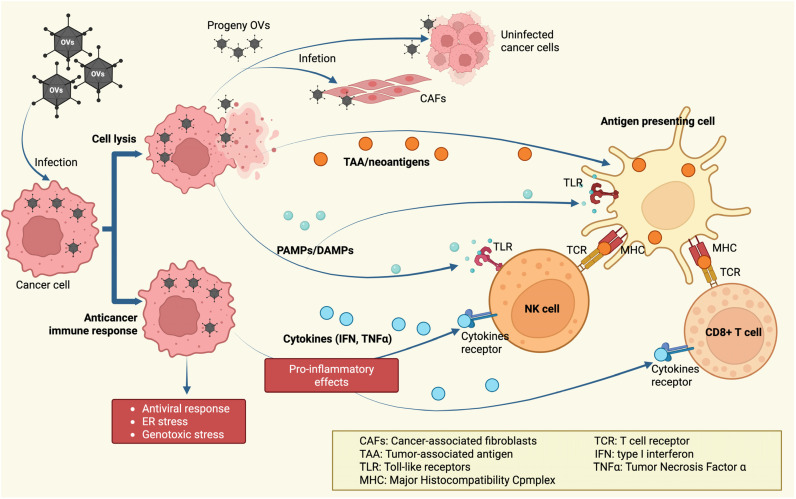
Mechanisms of action of oncolytic viruses in destroying cancer cells. After an oncolytic virus invades a cancer cell, it replicates until the cell is destroyed, releasing new viral particles that spread to neighboring malignant cells and decrease immunosuppressive populations such as cancer-associated fibroblasts (CAFs). As tumor cells break down, they release new viruses along with tumor-associated antigens (TAAs), including neoantigens, pathogen-associated molecular patterns (PAMPs), and danger-associated molecular patterns (DAMPs). APCs like dendritic cells engulf these released antigens and neoantigens, process them, and present them to the immune system, which enhances the expression of major histocompatibility complex (MHC) classes I and II on APCs and facilitates MHC–peptide–T-cell receptor interactions. PAMPs comprise viral particles, while DAMPs are host-derived proteins such as extracellular ATP, HMGB1, exposed calreticulin, and elevated heat shock protein 70. The combined presence of both PAMPs and DAMPs stimulates the maturation and activation of dendritic cells and triggers immune receptors such as TLRs on natural killer (NK) cells. Collectively, these processes enable immune responses against virus-infected cancer cells and foster recognition of TAAs and neoantigens on remaining tumor cells. Upon viral infection, cancer cells also mount an antiviral response characterized by endoplasmic reticulum (ER) and genotoxic stress, resulting in the production of antiviral cytokines. Among these, type I interferons (IFNs), are critical in recruiting and activating APCs, CD8^+^ T cells, and NK cells, creating a highly immunogenic and hostile environment for the tumor [66,92] (created with BioRender.com, accessed on 1 June 2025).

In addition to harnessing the natural lytic abilities of oncolytic viruses to eliminate cancer cells directly, the viral infection of these cells triggers a typical antiviral immune response aimed at eradicating the virus. This process is thought to revitalize or stimulate antitumor immune responses by shifting the tumor microenvironment from an immunosuppressive to an inflamed state. When a virus infects a tumor, there is an upsurge in pro-inflammatory cytokines that help recruit and activate both innate and adaptive immune cells [89]. The viral infection also triggers the release of strong immune-activating signals, such as Toll-like receptor ligands, which are essential for stimulating antigen-presenting cells (APCs), natural killer (NK) cells, and T lymphocytes [90] (Figure 3). The combined activity of these cytokines and TLR ligands helps counteract tumor-induced immune suppression. In addition, the virus’s lysis of cancer cells exposes and releases tumor antigens—including previously hidden or novel (neoantigenic) targets—to the immune system. This exposure occurs in the context of an inflammatory immune response, thereby enhancing overall antitumor immunity [91].

## 5. OVs—Genetically Modified

Genetic engineering provided a tool for creating viruses that (i) act selectively against cancer cells; (ii) have increased cytotoxic potential; (iii) stimulate the immune system; or (iv) modulate the tumor microenvironment. The tumor immunosuppressive barrier is a complex set of mechanisms that inhibit the immune system’s ability to mount an effective response against cancer cells. This barrier involves, among others, suppressor cells (regulatory T cells, myeloid suppressor cells), immunosuppressive cytokines (e.g., IL-10, TGF-beta), and enzymes such as indoleamine dioxygenase, which reduce the availability of tryptophan, a nutrient necessary for the activation of cytotoxic lymphocytes. Furthermore, the tumor may overexpress immune inhibitors such as PD-L1, which induce T cell anergy and silencing. This barrier protects the tumor from immune attack, enabling its growth and metastasis [93]. Therefore, reducing dominant immunosuppressive pathways improves effector cell recruitment, increases cytokine production, and restores the immune attack on the tumor [94].

One of the milestones in this research was the approval by the National Medical Products Administration (formerly the State Food and Drug Administration) of Adenovirus H101 as a therapeutic agent for the Chinese market, particularly for advanced nasopharyngeal carcinoma in combination with cisplatin and 5-FU chemotherapy [95]. Thanks to the deletion of the *E1B* gene, this virus lost the ability to inactivate the p53 protein and thus became susceptible to apoptosis in normal cells that produce p53. However, it could freely replicate in cancer cells, where the pathway regulating the synthesis of the pro-apoptotic p53 protein is almost always impaired. This mechanism was largely inspired by the first engineered adenovirus, ONYX-015, which showed promising results in Phase I and II clinical trials in the United States. However, progress in the field slowed after the death of a patient with an inborn error of metabolism (ornithine transcarbamylase deficiency), who received an adenoviral vector as part of a gene therapy trial. Although ONYX-015 was not responsible for this event—as the fatal reaction resulted from a different adenoviral vector used to treat the deficiency—the incident led to widespread concern. This event caused a delay in all gene therapy trials in the United States for some time and led to stricter safety regulations for future gene therapy research [95].

The first therapeutic based on an engineered oncolytic virus approved by the European Medicines Agency (EMA) and the Food and Drug Administration (FDA) in 2015 was T-VEC, which was authorized as a single agent for treating advanced melanoma. T-VEC is an engineered herpes simplex virus type 1 with three main modifications. It has two gene deletions: (i) of the ICP34.5 protein, a neurovirulence factor responsible for replication in normal cells, and (ii) of the ICP47 protein, which hinders recognition of infected cells by CD8^+^ T cells. Additionally, it includes the insertion of (iii) a human transgene encoding granulocyte-macrophage colony-stimulating factor (GM-CSF), a cytokine that attracts immune cells [96].

These modifications resulted in the development of an OV with high selectivity for cancer cells, which acts both directly through lytic activity and indirectly by recruiting immune-competent cells to the site of viral replication. Although the drug was approved for melanoma treatment, clinical trials are currently in progress to evaluate its effectiveness for other cancer types. Most recently, in 2025, a phase II clinical trial was initiated combining T-VEC with external beam radiation therapy to treat locally advanced soft tissue sarcoma (NCT06660810).

Adenoviruses are frequently engineered for cancer therapy due to their ability to accommodate large DNA inserts, enabling extensive genetic modification. A key adenoviral gene, *E1A*, overrides cell cycle control mediated by Rb protein, resulting in uninterrupted viral replication. Researchers have adapted *E1A* regulation to enhance the selective targeting of tumor cells. For instance, in vectors intended for prostate cancer treatment, *E1A* expression is driven by a prostate-specific antigen promoter—restricting viral replication and tumor cell destruction to prostate cells [97,98]. In other non-prostate cells, the absence of *E1A* expression means infection is halted as Rb-mediated apoptosis proceeds normally. Another engineered vector, KH 901, restricts *E1A* expression to dividing cells by combining the human telomerase reverse transcriptase promoter with an additional E2F-1 regulatory sequence [99]. This promoter combination broadens adenovirus targeting to a wider range of tumor types. *E1A* modulation is also used in oncolytic adenovirus CG0070, which selectively replicates in cells lacking functional Rb, a defect common in cancer. Under normal conditions, Rb binds to and inhibits E2F, blocking its transcriptional activity [100]. When Rb is mutated or absent—frequent in tumors—E2F-1 can activate the *E1A* gene directly, driving viral replication only in susceptible cancer cells [101]. The hypoxic environment often found in tumors makes them resistant to oxygen-dependent therapies like radiation. However, enhanced oncolytic adenoviruses can exploit these conditions; for example, HYPR-Ad is engineered to express *E1A* under HIF-1α control, a protein activated under hypoxic conditions, providing additional specificity for hypoxic tumor targeting [102].

Oncolytic viruses are engineered to kill cancer cells via both direct lysis and the use of included suicide genes. A common approach involves modifying adenoviruses to express the HSV-1 thymidine kinase (TK) gene. This strategy leverages the virus’s specificity for cancer cell targets, enabling a unique mechanism when combined with drugs like acyclovir or ganciclovir. HSV-1 TK differs from the human version in that it phosphorylates thymidine analogs to produce monophosphates, which are then sequentially phosphorylated by cellular kinases to form triphosphates. These active forms are incorporated into the DNA of dividing cells, ultimately causing DNA chain termination and cell death [103]. To improve targeting, tumor-specific promoters can be used to limit HSV-1 TK expression to specific tumor types—such as utilizing the osteocalcin promoter for bone metastasis targeting in clinical trials. Other viral suicide genes, like the adenovirus death protein, have also been introduced into OVs during preclinical research to further promote tumor cell killing [104,105]. By equipping OVs with suicide genes, their capacity to mediate direct cancer cell death is enhanced. Additional engineering strategies include the insertion of pro-apoptotic elements, for instance, those encoding TNF-related apoptosis-inducing ligand. While typically associated with apoptotic cell death, this molecule has recently been linked to necroptosis, a form of programmed necrosis, broadening the cytotoxic repertoire of OVs [106,107,108,109].

In addition to inducing standard antiviral immune responses, OVs are purposefully engineered to amplify the body’s immune attack on tumors. OVs can increase pro-inflammatory cytokine production, enhance antigen presentation in tumor cells, and induce tumor deaths that are more likely to activate the immune system [110]. Tumors often evade immunity by modifying the local microenvironment, recruiting suppressive cell types, and secreting cytokines that dampen immune activity. To overcome these barriers, OVs are genetically modified to disrupt the suppressive tumor microenvironment and favor immune-mediated tumor destruction. A notable example is T-VEC, which was designed to carry two copies of the human *GM-CSF* gene. GM-CSF cytokine can recruit and mature APCs—such as dendritic cells—promote antigen presentation, drive immune cell infiltration, enhance NK cell activity, and stimulate tumor-reactive T cells [82,91,111,112]. Analogous modifications have been introduced into adenoviral and vaccinia virus vectors [54,113,114]. Furthermore, some oncolytic vaccinia viruses have been armed with genes encoding interleukins like IL-7 and IL-24 to help overcome an immunosuppressive tumor microenvironment and resistance to therapy [114,115,116].

Adenoviruses have also been modified to enhance innate antigen presentation in cancer cells, often by driving the expression of heat shock protein 70. This strategy increases the transport of proteins to the proteasome, thereby generating more peptide fragments for presentation to the immune system. Heat shock protein 70 also facilitates antigen presentation by stabilizing peptide binding and promoting their recognition by APCs [117]. In addition, improving antitumor immunity often involves introducing costimulatory molecules, since cancer cells are typically deficient in these important immune-activating components [118]. Scientists have designed OVs to encode T-cell costimulatory proteins such as OX40, CD40, ICAM-1, B7-1, and LFA3, all of which can collectively strengthen immune responses to tumors [119,120,121]. For example, VALO-D102 is a novel adenoviral construct encoding CD40L and OX40L, which has demonstrated improved tumor control and increased infiltration of tumor-specific CD8^+^ T cells in melanoma models. When used with anti-PD-1 checkpoint blockade, VALO-D102 provided even stronger tumor suppression than either treatment alone [122]. Another modified adenovirus, LOAd 703, which encodes CD40L and 4-1BBL, has been shown to enhance cytotoxic T cell activity and inhibit tumor progression in a multiple myeloma xenograft model; it also increases tumor immunogenicity by upregulating costimulatory markers CD80 and CD86 [123,124].

Another method to enhance the effectiveness of OVs involves coating them with tumor-specific antigens to improve recognition and T-cell targeting. Ylösmäki and colleagues demonstrated that when adenoviruses are administered directly into tumors after being coated with modified tumor antigen peptides—sourced from proteins such as tyrosinase-related protein 2, human glycoprotein 100, melanoma-associated antigen A1, transmembrane and TPR repeat-containing protein 2, WD repeat domain 11, zinc finger RNA-binding protein, and a disintegrin and metalloproteinase with thrombospondin motifs 9—there is a marked increase in tumor-specific T cell responses. This approach not only limits tumor progression but also elicits a broad anticancer immune response systemically in both conventional and humanized mouse models of melanoma and triple-negative breast cancer [125].

Some oncoviruses have been found to encode cytokines, such as IL-12 (e.g., VVL-m12, HSV-1-IL12, VSV-mIL12, and SKV-012). This cytokine recruits cytotoxic lymphocytes, activates macrophages, and enhances the efficacy of PD-1/PD-L1 blockade therapy [126,127]. Furthermore, tumor expression of IL-12 increases local IFN-γ levels, promotes immunogenic cell death, and converts “cold” tumors into “hot” (immunologically active) ones [128]. In a phase I trial, patients with advanced solid tumors received intratumoral injections of escalating doses of SKV-012, a novel engineered oncolytic virus. This virus contains the viral neurovirulence gene *ICP34.5* driven by the Survivin promoter and includes an upstream genetic element for interleukin-12 expression. The study (NCT06080984) reported no dose-limiting toxicities and only mild adverse events. Among the participants, three showed partial tumor responses, one maintained stable disease, and two experienced disease progression. SKV-012 modified the tumor microenvironment by increasing infiltration of CD8^+^ T cells and conventional dendritic cells and by upregulating programmed death-ligand 1 expression [128].

OVs replication rates and oncolysis could also be improved by modifying genes involved in viral replication. For example, replacing the *P* gene of the MeV vaccine strain with that of the wild-type MeV (IC-B) can change the behavior of the engineered virus, aiding its spread throughout the tumor [129].

## 6. Next-Generation OVs

VCN-01 is a genetically engineered adenovirus serotype 5 designed to selectively replicate in tumor cells exhibiting disruptions in the retinoblastoma pathway, thereby sparing normal cells. The virus’s fiber protein has been modified by substituting its original heparan sulfate glycosaminoglycan-binding motif with an integrin-recognizing sequence, enhancing its affinity for cancer cells while minimizing liver cell tropism. Additionally, VCN-01 expresses the human sperm hyaluronidase (PH20), an enzyme that degrades components of the tumor extracellular matrix. This combination allows the virus to propagate through tumor masses via selective oncolysis and to facilitate viral dissemination and improved penetration of co-administered anticancer agents by enzymatically remodeling the tumor microenvironment (TME) [130]. VCN-01 is most often administered intravenously, providing effective access to solid tumors, and clinical studies have confirmed the virus’s presence in tumor tissue after a single infusion. This route of administration was chosen due to the difficult access to many solid tumors, such as pancreatic cancer, and its potential effectiveness against metastatic foci. This oncovirus is primarily active against solid tumors (pancreatic principally, central nervous system, and retinal tumors). Limited data also suggest a potential impact on metastases, especially in a diffuse tumor microenvironment. Side effects are usually mild (fever, flu-like symptoms, weakness, nausea) and, less frequently, more serious: thrombocytopenia, neutropenia, elevated liver enzymes, and, very rarely, enterocolitis or severe inflammatory reactions typical of high doses of adenoviruses [131]. In I phase clinical studies, this next-generation OV demonstrated a safe profile when administered intravenously or intratumorally with nab-paclitaxel plus gemcitabine, resulting in improved outcomes in patients with advanced ductal pancreatic adenocarcinoma [130,131].

EnAd (Enadenotucirev) is a chimeric group B adenovirus (derived from a combination of serotypes Ad11p and Ad3), designed as an oncolytic virus that selectively replicates and destroys cancer cells with minimal impact on healthy cells. The virus exhibits potent cytotoxicity against a variety of epithelial cancers and acts by rapidly destroying the tumor cell membrane, primarily through oncogenic mechanisms, releasing proinflammatory mediators, and inducing an anti-tumor immune response. EnAd is characterized by its ability to drive robust local transgene expression in tumor tissue and to stimulate dendritic cell maturation and T cell activation. It is primarily administered intravenously allowing it to reach many solid tumors and potentially micrometastatic foci, even in difficult-to-penetrate organs. The advantage of this route of administration is protection against rapid antibody-mediated neutralization (due to capsid modification) and the ability to target diffuse tumors [132]. EnAd primarily acts on solid tumors (colon, ovarian, pancreatic, and lung cancer). Its effectiveness against metastases is currently being investigated; an influx of immune cells and stabilization of the disease were observed in patients with metastases [133]. The most common side effects included mild flu-like symptoms, fever, fatigue, transient neutropenia, increased cytokines (IL-6, TNF-alpha), rash, and injection-site pain. Adverse reactions were usually transient and reversible [132]. EnAd is currently being intensively studied in several early-phase clinical trials (including immunotherapy), both intravenously and locally. Its mechanism allows it to be used as a vector for the delivery of other biological anticancer drugs, such as checkpoint-blocking antibodies [134,135].

DNX-2440 is a next-generation oncolytic adenovirus developed by DNAtrix. This virus selectively replicates in cancer cells and also encodes the human OX40 ligand (OX40L, CD252), which acts as a costimulator for T lymphocytes. During viral replication, OX40L is expressed on the surface of infected cancer cells, enhancing the local immune response against the tumor and promoting the survival of immune memory cells. It inhibits regulatory T cell activity in the tumor microenvironment. As a result, DNX-2440 not only destroys tumor cells through replication and lysis but also initiates an immune response, leading to a lasting anti-tumor memory, and the so-called abscopal effect has been observed in preclinical models. DNX-2440 is administered mainly intratumorally for the treatment of brain tumors (glioblastoma, metastatic liver tumors), as well as into the surgical space after resection, and sometimes into the cavities of solid tumors, allowing direct viral replication within the tumor mass and strong local immune activation (NCT03714334, NCT04714983) [136,137]. The safety profile of DNX-2440 is favorable, with mild flu-like symptoms—headache, nausea, fatigue, and transient fever—reported most often. No serious adverse events (grade 3/4) were reported in the latest Phase I/II studies. Local inflammation and temporary enzyme elevations were infrequently observed [138].

Delytact (teserpaturev; G47Δ) is a third-generation oncolytic virus developed from a genetically modified herpes simplex virus type 1 (HSV-1). It is the world’s first oncolytic virus approved for the treatment of glioblastoma—it has had conditional marketing approval in Japan for patients with malignant gliomas since 2021. The virus has three modifications: deletion of the *α47* gene and both copies of the *γ34.5* gene, and inactivation of the *ICP6* gene by insertion of the *lacZ* gene. This design ensures selective replication in cancer cells while losing the ability to replicate effectively in healthy neural tissue. Its mechanism of action involves both direct lysis of cancer cells by the replicating virus and stimulation of an antitumor response by inducing T lymphocytes that recognize tumor antigens. Intratumoral administration is clearly the dominant route, primarily into resected or remaining brain tumors. Direct administration enables extensive viral replication within the tumor, reduces the risk of systemic complications, and enables precise dosing in the challenging tumor microenvironment [139]. G47Δ is mainly aimed at eliminating solid tumors (gliomas, some central nervous system (CNS) tumors); however, clinical trials are underway to evaluate its use against metastases to the brain or other organs (although it is registered mainly for primary CNS tumors) [140]. The most common side effects associated with this oncovirus therapy include moderate fever, flu-like symptoms, headache, transient neurological disorders, cerebral edema, and seizures; less frequently, hematological disorders or infections. Most adverse effects (AEs) are transient and do not require discontinuation of therapy, and life-threatening complications are rare [139]. In clinical trials, G47Δ prolonged the survival of patients with relapsed or refractory brain tumors [139]. In a pivotal Japanese phase II study (19 patients), 1-year survival following Delytact administration was 84%, with a median overall survival of 20.2 months (range 16.8–23.6). This significantly exceeds the historical 1-year survival rate in glioblastoma, which is approximately 15% [141].

The most advanced oncolytic cowpox virus (CPXV) mutants are genetically modified variants that lack, among other things, the TK gene, thereby increasing selectivity for cancer cells and limiting infectivity in healthy tissues. Particularly promising are mutants armed with additional therapeutic genes, such as FCU1 encoding gene (an enzyme that converts the inactive prodrug 5-fluorocytosine to the cytotoxic 5-fluorouracil directly within the cancer cell) [142]. The virus causes direct lysis of tumor cells (oncolysis) and strongly stimulates the immune response to tumor antigens, thereby increasing the activation of T lymphocytes and dendritic cells. Additional expression of therapeutic genes encoding e.g., IFNγ enables local activation of prodrugs or enhancement of the immune response within the tumor [143]. CPXV is most frequently administered either intratumorally or systemically (intravenously) in preclinical and early clinical trials. Intratumoral delivery enables selective viral replication and strong immune activation within the tumor microenvironment, while systemic delivery is being evaluated for treating large tumors and possible metastases [142]. CPXV shows high activity against solid tumors (glioma, melanoma, colon cancer), however, in vivo models have also demonstrated an effect against micrometastases, especially after modifications with the insertion of transgenes increasing immunogenicity and cytotoxicity (e.g., FCU1) [142]. At very high systemic doses of CPXV, toxicity and bullous lesions were observed in mice (90% mortality at 10^7^ PFU/day). Modified thymidine kinase-deficient mutants showed a significant reduction in their ability to replicate in healthy tissues. They were well tolerated: mild local symptoms, fatigue, transient flu-like symptoms, and local bullous lesions. No serious adverse events were observed at therapeutic doses [144]. The latest available data on CPXV come from advanced in vitro and in vivo studies (animal models, xenografts), which confirm the high anti-cancer efficacy and safety of these constructs [142].

## 7. Routes of Administration of Oncolytic Viruses in Anticancer Therapies

Oncolytic viruses can be administered to patients by various routes, and the choice of administration method—and especially the relationship between local and systemic effects—is crucial in the context of immunotherapy.

### 7.1. Local Administration (Intratumoral)

Viruses are injected directly into the tumor, ensuring a high concentration of the virus and its proteins within the tumor. This promotes potent local activation of the immune system, including cytokine release, immunogenic cell death, and presentation of tumor antigens. Immunologically, local administration elicits a strong tumor response, minimizes systemic toxicity, and enables high local concentrations of immunomodulators or cytokines. This strategy helps convert a “cold” tumor into an immunologically “hot” one and activates lymphocytes locally. It also limits systemic exposure and lowers the risk of complications such as cytokine storms and autoimmunity [94,145].

### 7.2. Systemic Administration (Intravenous)

Systemic administration enables the virus to reach all tumor sites, including metastases. However, it also leads to rapid viral capture and neutralization by antibodies and phagocytic cells (liver and spleen), as well as a strong systemic response. This raises the risk of adverse effects (cytokine storm, toxicity), limits the duration of viral persistence, and often necessitates the development of armed or encapsulated viruses and immune cloaking strategies [146]. Intravenous administration of oncolytic viruses can control metastatic lesions (“abscopal effect”) by systemic activation of the anti-tumor response, but may be less effective in the presence of high innate or acquired antiviral immunity and may pose a higher risk of generalized immune toxicity [147]. The ultimate effect of systemic administration depends on the virus’s ability to bypass “immune traps” (e.g., neutralizing antibodies, hepatic macrophages) and to balance cytotoxicity with systemic immune activation [94].

## 8. Limitations of Oncolytic Virus Monotherapy

Oncolytic virus monotherapy faces challenges, including tumor heterogeneity, immune system interference, and difficulties achieving both local and systemic effects. These issues can diminish the therapy’s effectiveness and limit its ability to target and destroy cancer cells, especially in metastatic and solid tumors.

In solid tumors, oncolytic viruses are confronted with several barriers that limit their ability to reach and act on cancer cells efficiently. One of the primary hurdles is the physical barrier of the endothelial layer, which viruses must traverse to gain access to their targets within the tumor [148]. Beyond this, factors such as abnormal lymphatic drainage, heightened vascular permeability, and the densely packed extracellular matrix characteristic of solid tumors can all contribute to increased interstitial pressure, impeding viral penetration and distribution [149]. Furthermore, the presence of the virus inside the tumor can provoke a robust innate immune response, triggered by interactions with APCs. Complicating this further, pre-existing antiviral immunity, circulating neutralizing antibodies, and other blood components—such as coagulation factors FIX and FX and the complement protein C4BP—may neutralize or eliminate the virus before it can exert its therapeutic effects. Consequently, these host defense mechanisms reduce the fraction of virus that ultimately reaches and infects the tumor [149,150].

A significant barrier to the effectiveness of oncolytic viruses in solid tumors is the TME. Tumor cells in this environment can efficiently avoid immune detection, proliferate rapidly, and metastasize, particularly when immune regulation is compromised. Solid tumors secrete various chemokines and cytokines, including IL-10 and TGFβ, which dampen immune responses by suppressing immune cell populations and recruiting immunosuppressive cells such as T-regulatory cells, myeloid-derived suppressor cells, tumor-associated macrophages, tumor-associated fibroblasts, and neutrophils [151]. These components collectively shield tumor tissue from antitumor immune activity, impeding effective therapeutic responses [152]. As a result, inhibitory immune pathways and checkpoint molecules—such as PD-1, CTLA-4, TIM-3, and LAG-3—are often highly expressed on tumor-infiltrating lymphocytes, which further supports the development of an immunosuppressive tumor landscape.

Additionally, the abnormal architecture of tumor blood vessels can significantly restrict blood flow within tumors [148]. The resulting hypoxic and acidic microenvironment can inhibit tumor cell death, stimulate the formation of new blood vessels, increase growth factor production, and make cancer cells more resistant to therapies such as radiotherapy, chemotherapy, and immunotherapy [153,154]. For this reason, oncolytic viruses must preserve their activity within the immunosuppressive TME after reaching the tumor, as this niche critically affects cancer cell growth and survival.

## 9. Combined OV Therapies

Oncolytic viruses are increasingly studied for combination therapies to boost their ability to fight cancer. These viruses can specifically target and kill cancer cells while also eliciting an immune response. By combining OVs with treatments like chemotherapy, radiotherapy, immunotherapy, and targeted therapies, there is potential to overcome the limitations of OVs as a monotherapy and achieve stronger anti-tumor effects through synergy.

### 9.1. Combination of OVs with Radiotherapy or Chemotherapy

Radiotherapy is frequently used to treat localized tumors and early-stage cancers, but it has notable drawbacks, including non-specific effects on healthy tissue and the tendency for tumors to develop resistance to radiation, reducing its overall effectiveness. Metastatic cancers are particularly challenging, as radiotherapy typically fails to control disease at distant sites. Similarly, many chemotherapies lack selectivity, leading to damage in normal tissues and widespread toxicity, which can significantly affect the patient’s quality of life. Tumors may also evolve by acquiring mutations that confer drug resistance, thereby diminishing or negating the impact of chemotherapy [155]. By contrast, oncolytic viruses are able to infect and destroy malignant cells selectively. Unlike conventional therapies that are confined to regional application, oncolytic viruses have the unique advantage of targeting metastatic tumors, since they can spread systemically and infect tumor cells throughout the body. As a result, combining oncolytic viruses with chemotherapy or radiotherapy can potentially provide comprehensive tumor control at both local and distant site [156,157]. Clinical studies examining these combination approaches have often reported encouraging results with few side effects. Table 2 presents a selection of clinical trials that have evaluated oncolytic virus–chemoradiotherapy regimens.

### 9.2. Combination of Oncolytic Viruses and Immune Checkpoint Inhibitors

Combining oncolytic viruses with immune checkpoint inhibitors (ICIs) holds great promise for advancing cancer immunotherapy. Oncolytic viruses directly destroy malignant cells and stimulate robust immune responses, while ICIs lift inhibitory pathways that restrict the immune system, enabling immune cells to better recognize and eliminate cancer cells. This combination produces a synergistic effect that could enhance outcomes in a range of cancer types. Common ICIs used in this context include Nivolumab, Ipilimumab, Pembrolizumab, and Atezolizumab.

ICIs work by amplifying T cell-mediated anti-tumor activity by reversing immunosuppression within the tTME. Oncolytic viruses can trigger immune activation and increase infiltration of immune cells into tumors; ICIs further potentiate this effect by antagonizing inhibitory checkpoints, thereby promoting immune cell function and improving therapeutic responses. The efficacy of ICIs is highly dependent on the TME: tumors designated as “hot”—those with more immune infiltration—are more responsive, while “cold” tumors exhibit poor response. Therefore, strategies that alter and improve the TME are vital for boosting treatment outcomes [158].

Numerous clinical trials have been conducted on combination therapies with OVs and ICIs, some of them are presented in Table 3.

### 9.3. Combination of Oncolytic Viruses with CAR-T Cells

CAR-T cells are patient T cells engineered to express chimeric antigen receptors that recognize specific antigens on the surface of cancer cells. This is an advanced form of immunotherapy that harnesses the patient’s own immune system to fight cancer, particularly in cancers that are resistant to standard treatment [159]. However, CAR-T cells show limited efficacy in solid tumors; therefore, new strategies are under development to enhance tumor infiltration, persistence, and overall function, e.g., by expressing chemokine receptors that promote T cell infiltration or by genetically modifying CAR-T cells to secrete immune-modulating compounds to overcome TME immune suppression [160].

In this context, combining oncolytic viruses with CAR-T cells presents a promising approach in cancer immunotherapy. This strategy aims to harness the strengths of both methods: CAR-T cells’ targeted destruction of cancer cells and oncolytic viruses’ ability to stimulate anti-tumor immune responses and alter the TME. Preclinical evidence strongly suggests that this combination therapy has significant potential for cancer treatment (Table 4).

**Table 4:** Examples of preclinical studies on oncolytic viruses and CAR-T cells.

Oncolytic Virus	Armed	Effect of OV	References
Adenovirus	Onc.—Ad RANTES IL-2	Unblocking the tumor microenvironment for CAR-T (RANTES chemokine) and improving their survival/effector function and reducing systemic toxicity (local IL-2 effect)	[161]
Adenovirus	CAdvec-αPDL1	Local, specific reversal of tumor microenvironment immunosuppression by blockade of the PD-1/PD-L1 axis without systemic effects.	[162]
Adenovirus	CAdvec-IL12 αPDL1	Local, specific reversal of tumor microenvironment immunosuppression by blockade of the PD-1/PD-L1 axis without systemic effects, potent activation and proliferation of effector lymphocytes within the tumor via IL-12.	[163]
Adenovirus	oAD-IL7, B7H3-CAR-T	Significantly increased survival, proliferation, and tumor abundance of CAR-T lymphocytes, resulting in a more potent and long-lasting antitumor effect.	[164]
Adenovirus	CAd12_PDL1	Local, specific reversal of tumor microenvironment immunosuppression by blockade of the PD-1/PD-L1 axis without systemic effects, potent activation and proliferation of effector lymphocytes within the tumor via IL-12.	[165]
Vaccinia virus	VSVmIFNβ EGFRvIII CAR T	Dual T cell activation—by tumor antigen (EGFRvIII) and by viral antigen VSV—and potent “reprogramming” of the tumor microenvironment—eliminating both the primary tumor and distant metastases.	[166]
Vaccinia virus	CAR/CXCL11 VV. CXCL11	Significant increase in CAR-T influx and infiltration into the tumor due to CXCL11 secretion, transforming a “cold” tumor into a “hot” one.	[167]
Herpes virus	CAR-T/A56 antigen	Introduction of the A56 antigen into the tumor using an oncolytic virus enables highly selective and effective CAR-T therapy, even in areas where classic tumor target antigens are absent, and the side effects on healthy tissue are minimal.	[168]
Herpes virus	oHSV T7011 with CD19 or BCMA CAR T-cell	oHSV T7011 infects tumor cells and forces them to express “artificial” CAR antigens such as CD19 and BCMA, making them targets for conventional CAR-T.	[169]
Herpes virus	oHSV-1 CD70 CAR-T	The combination of oHSV-1 and CD70 CAR-T provides mutual enhancement of both therapies—enhancing CAR-T infiltration, activation, and survival, and “reprogramming” the tumor microenvironment from immunosuppressive to proinflammatory.	[170]
Pox virus	CD19-CAR-T	Introduction of the CD19 antigen into solid tumor cells that do not naturally express this marker, enabling the targeting of CD19-CAR-T cells (developed for the treatment of hematological malignancies) against solid tumors as well.	[171]

OVs can enhance CAR-T cell function in several ways: (i) they can be engineered to carry therapeutic transgenes that boost CAR-T activation; (ii) they have the potential to persist and maintain their cytotoxic activity within an immunosuppressed tumor microenvironment, thereby providing danger signals that may counteract tumor-induced immunosuppression; and (iii) their direct lytic activity on cancer cells causes tumor cell death, leading to the release of tumor-associated antigens [172].

**Figure 4:**
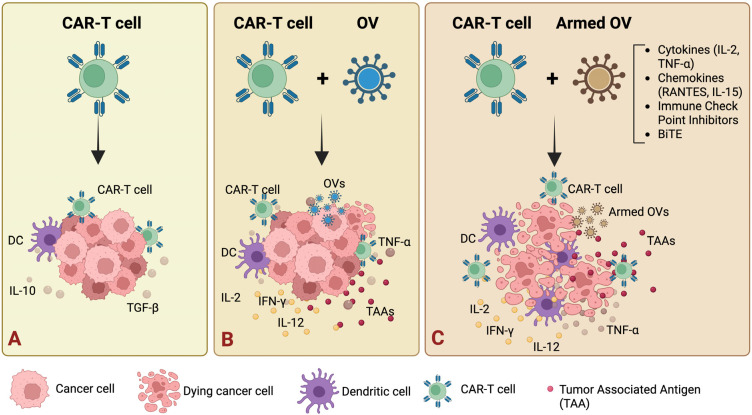
Combining CAR-T cells with oncolytic viruses presents a promising strategy for treating solid tumors. (**A**) CAR-T cells face significant hurdles in solid tumor environments—including immunosuppression—which can lead to T cell exhaustion and reduced therapeutic efficacy. (**B**) Delivering oncolytic viruses prior to CAR-T cell therapy can reduce tumor mass, enhance immunogenic cell death, and help to reverse local immunosuppression. (**C**) Oncolytic viruses can be engineered to introduce therapeutic genes into the tumor microenvironment, thereby augmenting T cell effector functions. Preclinical models have shown that combining CAR-T cells with oncolytic viruses engineered to express cytokines, chemokines, bispecific T cell engagers (BiTEs), or immune checkpoint inhibitors can significantly enhance antitumor effectiveness compared to either therapy alone [151,160] (created with BioRender.com, accessed on 1 July 2025).

A notable example of therapeutic synergy is combining CAR-T cell therapy with engineered oncolytic viruses that deliver chemokines such as RANTES and IL-15, which improve CAR-T cell trafficking, persistence, and antitumor activity [173] (Figure 4). For instance, using oncolytic vaccinia virus encoding CD19t can induce CD19 expression on tumor cells, sensitizing them to CD19-targeted CAR-T cells for enhanced lysis. Co-culturing these CD19 CAR-T cells with OV19t not only promotes cytokine release but also demonstrates robust killing of infected cancer cells [171]. Similarly, arming vaccinia virus with CXCL11—a CXCR3 ligand—was found to improve the migration of transferred effector cells into the tumor [167]. Another study showed that expression of a bispecific T cell engager by an oncolytic adenovirus targeting a secondary tumor antigen could effectively address antigen heterogeneity [174]. Other studies have explored expressing a bispecific T cell engager from oncolytic adenoviruses to target cancer cell variability in antigen display better, allowing activation of non-engineered T cells or those lacking the primary CAR target. In another strategy, an oncolytic adenovirus, combined with a helper-dependent vector encoding a PD-L1-blocking mini-antibody, helped overcome T cell exhaustion and enabled effective tumor cell targeting by CAR-T cells [162].

Furthermore, DD7-IL7 and B7H3 CAR-T cells, when tested together, showed enhanced proliferation and persistence of CAR-T cells in glioblastoma models [164]. The oncolytic adenovirus LOAd703, which expresses CD40L and 4-1BBL, has shown promise in B cell malignancies by activating antigen-presenting cells and T cells through CD46 engagement. When used in conjunction with CAR-T cell therapy, LOAd703 can elicit strong anti-lymphoma immune responses and significantly enhance the therapeutic impact of CAR-T cells [124].

Oncolytic vaccinia viruses engineered to secrete CXCL11 or express IL-21 have shown considerable potential to boost adaptive T cell transfer and promote vaccine-based immunotherapy, providing valuable new strategies to enhance treatment efficacy [167]. In addition, oncolytic viruses can be further modified to present specific CAR antigens on the tumor surface, thereby improving the targeting and effectiveness of CAR-T cell therapies [175]. Altogether, integrating oncolytic virotherapy with CAR-T cell approaches is a promising direction for the future of cancer treatment.

An interesting modeling analysis by Conte et al., based on in vitro studies (combining IL-13Rα2-targeted CAR T-cells with an oncolytic virus, HSV-based C134), showed that the virus burst size is a critical factor in determining the net tumor infection rate and the overall efficacy of the combined treatment in glioblastoma. Additionally, the model predicted that administering the oncolytic virus simultaneously with, or prior to, CAR T-cells could maximize therapeutic effectiveness [176].

A Phase 1 clinical trial is currently underway, in which the VCN-01 virus is administered intravenously, followed by an infusion of autologous CAR-T anti-mesothelin (huCART-meso) in patients with pancreatic and ovarian cancer (NCT03740256). Interim results for three patients with epithelial ovarian and pancreatic cancer showed that in two patients with measurable disease (both with ovarian cancer), the most significant decrease in disease volume was observed: 15% and 29.1% respectively, according to RECIST criteria. Adverse events of this therapy included cytokine release syndrome in one patient, as well as lymphopenia and elevated AST values, which were expected due to the use of VCN-01. No neurotoxicity was observed [177]. The most important clinical aspect is the disruption of the immunosuppressive tumor microenvironment—the oncolytic virus VCN-01 degrades the stromal barrier, facilitates CAR-T infiltration, and induces cytokine “flare.”

There are undoubtedly several benefits to combining CAR-T therapy with oncolytic viruses. OVs can help overcome the immunosuppressive microenvironment of solid tumors, which often hinders CAR-T cell function. They can do this by inducing immunogenic cell death, releasing tumor antigens, and creating an inflammatory environment that promotes T cell activation [151]. By creating a more favorable tumor microenvironment, OVs can enhance CAR-T cells’ ability to reach and survive within the tumor [168]. Additionally, by improving the tumor microenvironment, OVs can further enhance the effectiveness of CAR-T cells [160]. OVs can be engineered to express tumor-associated antigens, potentially broadening the range of antigens targeted by the immune system, including CAR-T cells [168]. According to Zhang et al., some studies have explored the use of CAR-T cells to deliver OVs systemically, which could potentially reach distant tumors that are difficult to access with localized OV injections [168]. On the other hand, the high cost of both CAR-T cell therapy and OVs, along with concerns about potential side effects, are significant obstacles to widespread clinical use [175].

## 10. Conclusions and Future Directions

Over the past decade, oncolytic virotherapy has rapidly progressed, with its primary goal shifting from merely causing direct tumor cell lysis to harnessing and enhancing virus-induced anti-tumor immune responses. The clinical approval of T-VEC for the intratumoral treatment of unresectable metastatic melanoma has established oncolytic viruses as a credible therapeutic option, especially for tumors that are resistant to chemotherapy, radiotherapy, targeted agents, or immunotherapy. These viruses provide versatile platforms for developing and improving targeted cancer treatments that destroy malignant cells through various mechanisms, thereby reducing the risk of therapeutic resistance.

Current efforts in oncolytic virotherapy research focus on improving delivery methods, enhancing safety, and developing next-generation viruses that specifically target cancer cells while sparing healthy tissue. There is a strong emphasis on combining oncolytic viruses with immune checkpoint inhibitors and other immunomodulatory agents, as these combinations have demonstrated synergistic effects that boost the effectiveness of virotherapy, especially for solid tumors. The integration of oncolytic viruses with CAR-T cell therapies is also a promising area, leveraging the strengths of each approach to combat tumor immune evasion. With ongoing progress and numerous clinical trials in progress, this combined immunotherapy approach may soon become a fundamental part of modern cancer treatment.

The need for effective metastatic targeting, personalization, and synergy with other immunotherapies shapes the future of oncolytic virus research. Strategies include advanced genetic engineering, enhancing tropism for metastatic or microenvironment-specific cells (e.g., by introducing ligands for receptors overexpressed in metastases), and novel therapeutic combinations involving checkpoint inhibitors, CAR-T technologies, or other immune-modulating agents. These advances are expected to allow for more precise and effective eradication of cancer metastases.

Among the currently studied platforms, HSV-1 (like T-VEC and OH2), adenoviruses (armed with PD-L1 inhibitors, IL-12, or other cytokines), poxviruses (such as VACV and CPXV), and VSV are especially promising. HSV-1 stands out for its engineering versatility in solid tumors; adenoviruses serve as potent immune “ignitors”; and poxviruses, such as VSV, offer strong immunogenicity and rapid replication. The most significant potential for therapeutic breakthroughs lies in combining oncolytic viruses with immune checkpoint blockades (PD-1, CTLA-4), CAR-T strategies to enhance T cell infiltration and reduce immunosuppression, or gene transfer of cytokines, chemokines, or ECM-cleaving enzymes. These next-generation strategies—including viruses specifically designed to convert “cold” tumors and multifocal metastases into immunologically “hot” and attackable lesions—are predicted to drive the future success of oncolytic virotherapy.

## Figures and Tables

**Table 2:** Clinical studies on oncolytic viruses used with radiation and chemotherapy treatments.

Oncolytic Virus	Cancer	Clinical Phase/Status	Therapy	Trail No.
AdV (Enadenotucirev)	Rectal Cancer	Phase 1/completed	radiotherapy	NCT03916510
Herpes simplex virus (GM-CSF)	Melanoma Stage IV	Phase 1/completed	radiotherapy	NCT05068453
Ad (ADV/HSV-tk)	Metastatic Non-small Cell Lung Cancer/Metastatic Triple-negative Breast Cancer	Phase 2/completed	radiotherapy	NCT03004183
Ad (NSC-CRAd-S-p7)	Glioma	Phase 1/completed	radiotherapy/chemotherapy	NCT03072134
Vaccinia virus (GL-ONC1)	Head and Neck Cancer	Phase 1/completed	radiotherapy/chemotherapy	NCT01584284
Ad (Ad-39yCD/mutTKSR1rep-ADP)	Non-small Cell Lung Cancer Stage I	Phase 1/completed	radiotherapy	NCT03029871
HSV (G207)	Recurrent/progressive pediatric high-grade gliomas	Phase 2/ongoing	radiotherapy	NCT04482933
Ad (LOAd703)	Pancreatic Cancer	Phase 1/2/ongoing	chemotherapy	NCT02705196
Ad 5 VCN-01	Pancreatic Cancer	Phase 1/completed	chemotherapy	NCT02045602 NCT02045589
Vaccinia virus (KM1)	Ovarian Cancer	Phase 1/ongoing	chemotherapy	NCT05684731
Vaccinia virus (TG6002)	Glioblastoma	Phase 1/2/completed	chemotherapy	NCT03294486
Vaccinia virus (GL-ONC1)	Ovarian Cancer	Phase 1/2/completed	chemotherapy	NCT02759588
HSV-2 (OH2)	Melanoma	Phase 3/ongoing	chemotherapy	NCT05868707
Measles virus (MV-NIS)	Ovarian/ Peritoneal Cancer	Phase 2/ongoing	chemotherapy	NCT02364713
HSV-1 (HF10)	Pancreatic Cancer	Phase 1/ongoing	chemotherapy	NCT03252808
Vaccinia virus (GL-ONC1)	Ovarian Cancer	Phase 3/ongoing	chemotherapy	NCT05281471
Herpes simplex 1 virus (Talimogene laherparepve)	Triple Negative Breast Cancer	Phase 1/2/completed	chemotherapy	NCT02779855
Vaccinia virus (Pexa-Vec)	Hepatocellular Carcinoma	Phase 3/completed	chemotherapy	NCT02562755
Reovirus (REOLYSIN)	Metastatic Colorectal Cancer	Phase 1/completed	chemotherapy	NCT01274624
Oncolytic virus VRT106	Pancreatic cancer	Phase 1/ongoing	chemotherapy	NCT06866977

**Table 3:** Clinical studies involving oncolytic viruses combined with immune checkpoint inhibitors.

Oncolytic Virus	Cancer	Clinical Phase/Status	Combination Drug Effect	Trail No.
RT-01	Advanced Solid Tumor	Phase 1/completed	Nivolumab & ANTI-PD-1	NCT05228119 NCT05122572
HSV-1 (RP1)	Solid tumors	Phase 1/2/completed	Nivolumab & ANTI-PD-1	NCT03767348
HSV-1 (RP3)	Squamous Cell Carcinoma of Head and Neck Hepatocellular Carcinoma	Phase 2/ongoing Phase 2/ongoing	Nivolumab & ANTI-PD-1 Atezolizumab & ANTI-PD-L1	NCT05743270 NCT05733598
HSV-1 (HF10)	Melanoma Stage III/IV Malignant Melanoma	Phase 2/completed Phase 2/completed	Ipilimumab & ANTI-CTLA4 Ipilimumab &ANTI-CTLA4	NCT03153085 NCT02272855
HSV-2 (OH2)	Melanoma	Phase 1/2/completed	Pembrolizumab & ANTI-PD-1	NCT04386967
Ad (DNX-2401)	Glioblastoma/Gliosarcoma	Phase 2/completed	Pembrolizumab & ANTI-PD-1	NCT02798406
Ad (TILT-123)	Ovarian Cancer Solid Tumor	Phase 1/ongoing Phase 1/ongoing	Pembrolizumab & ANTI-PD-1 Avelumab & ANTI-PD-1	NCT05271318 NCT05222932
EnAd (Ad11/Ad3)	Epithelial tumor	Phase 1/completed	Enadenotucirev & nivolumab	NCT02636036
Vaccinia virus (TBio-1)	Solid Tumor	Phase 1/2/completed	Pembrolizumab & ANTI-PD-1	NCT04301011
HSV (M032)	Glioblastoma Multiforme	Phase 1/2/ongoing	Pembrolizumab & ANTI-PD-1	NCT05084430
Reovirus (REOLYSIN)	Pancreatic Adenocarcinoma	Phase 1/completed	Pembrolizumab & ANTI-PD-1	NCT02620423
Ad (LOAd703)	Pancreatic Cancer Malignant Melanoma	Phase 1/2/ongoing Phase 1/2/completed	Atezolizumab & ANTI-PD-L1 Atezolizumab & ANTI-PD-L1	NCT02705196 NCT04123470
Ad (H101)	Recurrent Cervical Cancer Bladder Cancer	Phase 2/ongoing Phase 2/ongoing	Camrelizumab & ANTI-PD-1 Camrelizumab & ANTI-PD-1	NCT05234905 NCT05564897
Reovirus (PeLareorEp)	Breast Cancer Metastatic	Phase 2/completed	Avelumab & ANTI-PD-L1	NCT04215146
Vaccinia virus (BT-001)	Metastatic/Advanced Solid Tumors	Phase 1/2/ongoing	Pembrolizumab & ANTI-PD-1	NCT04725331
Coxsackie virus (A21)	Uveal Melanoma	Phase 1/completed	Ipilimumab & ANTI-CTLA4	NCT03408587
MG1-MAGEA3	Non-Small Cell Lung Cancer	Phase 1/2/completed	Pembrolizumab & ANTI-PD-1	NCT02879760
MEDI5395	Advanced Solid Tumors	Phase 1/completed	Durvalumab & ANTI-PD-L1	NCT03889275
Vaccinia virus (Pexa-Vec)	Refractory Colorectal Cancer	Phase 1/2/completed	Durvalumab & ANTI-PD-L1 Tremelimumab & ANTI-CTLA4	NCT03206073

## Data Availability

No new data were created in this study.

## Abbreviations

OV—oncolytic virus; HSV—herpes simplex virus; T-VEC—Talimogene laherparepwek; oAd—oncolytic adenovirus; CAR-T—chimeric antigen receptor T; Rb—retinoblastoma; VV—vaccinia virus; TK—thymidine kinase; RV—reovirus; NDV—Newcastle disease virus; MeV—measles virus; VSV—vesicular stomatitis virus; NK—natural killer; APC—antigen-presenting cell; GM-CSF—granulocyte-macrophage colony-stimulating factor; TME—tumor microenvironment; ICI—immune checkpoint inhibitor; EnAd—Enadenotucirev; CPXV—oncolytic cowpox virus.
